# YOU MADE IT TO A BIG NUMBER”: PERCEPTIONS OF OLDER ADULTHOOD AMONG ADULTS WITH SICKLE CELL DISEASE

**DOI:** 10.1093/geroni/igae098.3770

**Published:** 2024-12-31

**Authors:** Elizabeth Linton, Joseph Gallo, Jennifer Schrack, Lydia Pecker, Sophie Lanzkron

**Affiliations:** Johns Hopkins Bloomberg School of Public Health, Baltimore, Maryland, United States; Johns Hopkins Bloomberg School of Public Health, Baltimore, Maryland, United States; Johns Hopkins Bloomberg School of Public Health, Baltimore, Maryland, United States; Johns Hopkins School of Medicine, Baltimore, Maryland, United States; Sidney Kimmel Medical College, Philadelphia, Pennsylvania, United States

## Abstract

This study categorizes how adults with sickle cell disease (SCD) define older adulthood and identifies topics important for future gerontology research. Individuals aged 18 and older were recruited from a pilot mixed-methods study in an outpatient SCD clinic, selected for representation across age, sex, genotype, and frailty (physically robust, prefrail, frail) according to the 5-item FRAIL scale. Interviews were analyzed using Lieblich et al’s interpretive categorical-content approach. Five thematic categories defining the onset of older adulthood emerged: (1) SCD’s cumulative impact, (2) Observed lifespan of others with SCD, (3) Social determinants of health and lifestyle, (4) Statistical life expectancy, and (5) Social roles and transitions. Proposed research to promote healthier aging were (1) studying SCD sequelae and age-related comorbidities, (2) developing new SCD treatments and cure, and (3) identifying non-opioid pain management. Population aging for people living with SCD presents novel opportunities to promote dignity across the lifespan. This study’s findings may inform the content of health communications to facilitate goal-concordant SCD care.

